# Enzyme therapy in a structural light

**DOI:** 10.1107/S2052252526001557

**Published:** 2026-02-24

**Authors:** Jon Cooper

**Affiliations:** a Division of Medicine University College London London United Kingdom

**Keywords:** l-asparaginases, enzyme therapy, leukemia, reaction mechanisms

## Abstract

The paper by Gilski *et al.* in this issue [*IUCrJ* (2026), **13**, 132–145] focuses on the stereochemical analysis of the catalytic mechanism of asparaginases with the aim of resolving key ambiguities in the mechanism. These enzymes have found truly remarkable therapeutic success in the treatment of childhood leukemias.

The enzyme l-asparaginase (EC 3.5.1.1) catalyses the hydrolysis of the amino acid asparagine to aspartic acid and ammonia. Many asparaginases also have activity on glutamine, producing glutamic acid instead, along with ammonia. In the current classification of these enzymes they are grouped into three classes, the first of which is formed mainly by the bacterial asparaginases that are divided into two types. Type I asparaginases are cytosolic, have relatively low affinity for substrate and low glutaminase activity. In contrast, type II enzymes are usually periplasmic, have µ*M* substrate affinities and have comparable asparaginase and glutaminase activities (Boyd & Phillips, 1971 ▸; Chohan & Rashid, 2013 ▸; Davidson *et al.*, 1977 ▸). Type I and type II asparaginases have low sequence similarity, for example those from *Escherichia coli* have a sequence identity of only 24%. Generally, the enzymes form dimers or tetramers with a subunit molecular mass of 35 kDa (Fig. 1 ▸).

Biologically, the asparaginases have a wide range of roles. For instance, plants transport nitro­gen in the form of l-asparagine from their roots to growing tissues and thus have a high requirement for this enzyme (Atkins *et al.*, 1975 ▸; Sieciechowicz *et al.*, 1988 ▸). In bacteria, when amino acids become the primary carbon source in anaerobic conditions, the expression level of asparaginase can increase by 100-fold (Cedar & Schwartz, 1967 ▸; Cedar & Schwartz, 1968 ▸). This is important since the metabolites of asparagine (and glutamine) can feed into the citric acid cycle. In contrast, the preferred carbon source glucose is a catabolite repressor of asparaginase expression. Thus asparaginases and glutaminases are necessary for cell growth in ammonia-deficient media and their expression is activated by the presence of these amino acids in the medium.

Intriguingly, the type II asparaginases have been widely used as very effective chemotherapy treatments for acute lymphoblastic leukaemia (ALL), lymphoblastic lymphoma (LBL) and other hematopoietic malignancies. ALL is the most common childhood acute leukaemia, constituting approximately 80% of childhood leukaemias and 20% of adult leukaemias (Fullmer *et al.*, 2010 ▸). The history of asparaginase use for ALL treatment can be traced back to the 1950s, when it was found that the progression of murine lymphoma was reduced by guinea pig serum and the active component was a protein (Kidd, 1953 ▸). Later in the 1950s it was shown that a transplantable rat carcinoma cell line had an absolute requirement for asparagine (Neuman & McCoy, 1956 ▸). In the early 1960s, it was found that it was the asparaginase in guinea pig serum that accounted for its observed anti-lymphoma activity (Broome, 1963*a* ▸; Broome, 1963*b* ▸).

By 1964, it had been demonstrated that asparaginase from the bacterium *E. coli* had the same antitumor effect as guinea pig serum (Mashburn & Wriston, 1964 ▸). The larger quantities of asparaginase that could be produced from bacteria such as *E. coli* and *Erwinia chrysanthemi* allowed a series of preclinical and clinical studies of the enzyme as an infused drug. The success of this work culminated in widespread therapeutic use of the enzyme from the 1970s onwards as well as the development of a polyethyl­ene glycol modified version (PEG-asparaginase), which has improved stability and reduced immunogenicity. The effectiveness of asparaginase in the treatment of ALL was demonstrated beyond doubt (Hill *et al.*, 1967 ▸; Oettgen *et al.*, 1967 ▸). In recent studies, treatment with asparaginase has been shown to improve event-free survival for ALL from typically less than 10% to over 80% (Möricke *et al.*, 2008 ▸; Pui *et al.*, 2009 ▸; Silverman *et al.*, 2001 ▸).

The high demand for exogenous asparagine by leukemic lymphoblasts (Haskell & Canellos, 1969 ▸; Prager & Bachynsky, 1968 ▸) is due to their low levels of the enzyme asparagine synthetase, which is responsible for endogenous asparagine synthesis (Kiriyama *et al.*, 1989 ▸). Consequently the tumour cells can only obtain this amino acid from the blood stream, while healthy cells are not affected by asparaginase treatment because they possess sufficient asparagine synthetase to synthesize enough of it themselves.

This clinical use of an enzyme represents a truly remarkable approach for treatment of neoplastic, or indeed any, disease. The enzyme is also used commercially in the food industry since treatment of foods with l-asparaginase prior to cooking significantly reduces formation of the neurotoxin acryl­amide (Friedman, 2003 ▸).

The paper by Gilski *et al.* (2026 ▸) in the current issue of *IUCrJ* focuses on the stereoelectronic analysis of the catalytic mechanism of asparaginases based upon the hundreds of crystal structures of this enzyme from all three classes which are now available, although these were filtered down to a defined set of substrate or product complexes. The mechanism involves a β-acyl-enzyme, which is formed by a nucleophilic threonine residue attacking the amide carbon of the substrate asparagine. This is followed by the nucleophilic attack of the intermediate by a water molecule, which releases the product, l-aspartate (Verma *et al.*, 2007 ▸). Even in the best-studied class I enzymes there is some ambiguity as to the nature of this nucleophilic group, since there are two conserved and suggestively positioned threonine side chains in the active site. Similar ambiguity exists for the other classes, where metal ions are also likely to be involved. The paper concludes that Thr12, rather than Thr89, in *E. coli* type II asparaginase is likely to be the nucleophilic group and extends this analysis to the other classes and types.

These mechanistic studies relied on creation of a specialized database of corroborated asparaginase structures (Wlodawer *et al.*, 2024 ▸) and as such will assist greatly in fundamental studies of the enzyme. Indeed, who knows what the future may hold for further engineered forms of asparaginase with improved catalytic efficiency, stability and humanization.

## Figures and Tables

**Figure 1:**
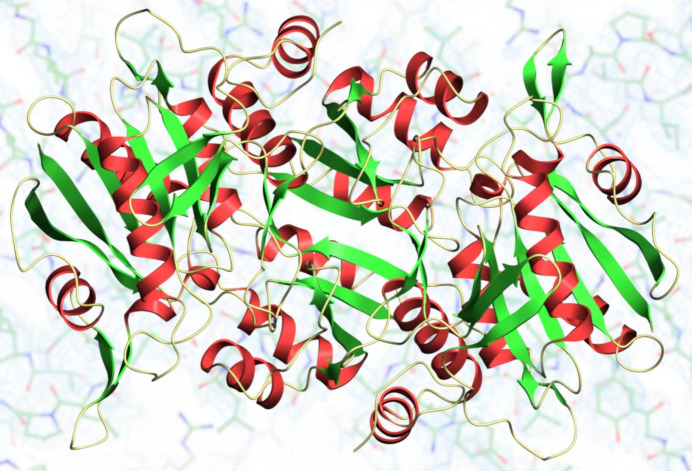
A dimer of the thermostable l-asparaginase from *Thermococcus kodakarensis* (RCSB extended ID: pdb_00005ot0; Guo *et al.*, 2017 ▸).
